# Preparation, Characteristics, and Advantages of Plant Protein-Based Bioactive Molecule Delivery Systems

**DOI:** 10.3390/foods11111562

**Published:** 2022-05-26

**Authors:** Tongwei Guan, Zhiheng Zhang, Xiaojing Li, Shaoning Cui, David Julian McClements, Xiaotian Wu, Long Chen, Jie Long, Aiquan Jiao, Chao Qiu, Zhengyu Jin

**Affiliations:** 1 College of Food & Bioengineering, Xihua University, Chengdu 610039, China; guantongweily@163.com (T.G.); wuxiaotian0421@163.com (X.W.); 2 State Key Laboratory of Food Science and Technology, School of Food Science and Technology, Collaborative Innovation Center of Food Safety and Quality Control in Jiangsu Province, Jiangnan University, Wuxi 214122, China; 6200112177@stu.jiangnan.edu.cn (Z.Z.); longchen@jiangnan.edu.cn (L.C.); jielong@jiangnan.edu.cn (J.L.); aqjiao@jiangnan.edu.cn (A.J.); phdqiu@jiangnan.edu.cn (C.Q.); 3 College of Light Industry and Food Engineering, Nanjing Forestry University, Nanjing 210037, China; lxj0810jy@163.com; 4 Department of Food, Yantai Nanshan University, Yantai 264005, China; csn0701@163.com; 5 Department of Food Science, University of Massachusetts, Amherst, MA 01060, USA; mcclemen@umass.edu

**Keywords:** plant protein, bioavailability, delivery, bioactive substance

## Abstract

As a renewable resource, the market trend of plant protein has increased significantly in recent years. Compared with animal protein, plant protein production has strong sustainability factors and a lower environmental impact. Many bioactive substances have poor stability, and poor absorption effects limit their application in food. Plant protein-based carriers could improve the water solubility, stability, and bioavailability of bioactive substances by different types of delivery systems. In this review, we present a detailed and concise summary of the effects and advantages of various plant protein-based carriers in the encapsulation, protection, and delivery of bioactive substances. Furthermore, the research progress of food-grade bioactive ingredient delivery systems based on plant protein preparation in recent years is summarized, and some current challenges and future research priorities are highlighted. There are some key findings and conclusions: (i) plant proteins have numerous functions: as carriers for transportation systems, a shell or core of a system, or food ingredients; (ii) plant protein-based carriers could improve the water solubility, stability, and bioavailability of bioactive substances by different types of delivery systems; and (iii) plant protein-based carriers stabilize bioactive substances with potential applications in the food and nutrition fields.

## 1. Introduction

**Figure 1:**
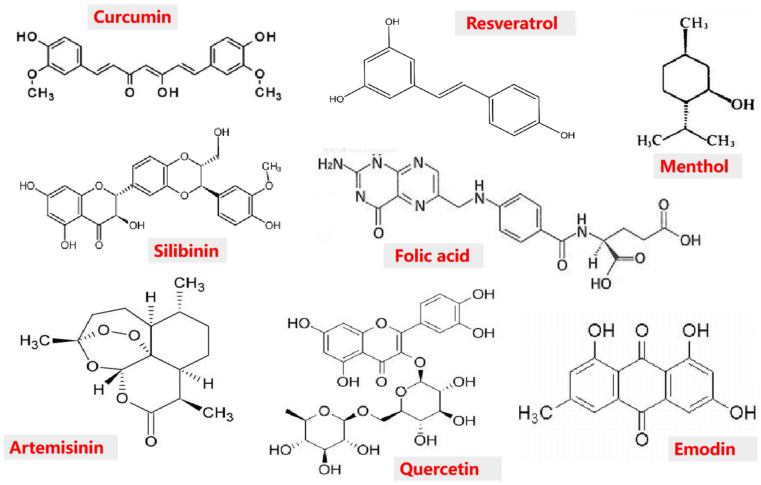
Molecular structure of some common bioactive substances.

With the development of the economy and society, there has been an increasing demand for improving human health through diet. Based on this, people have risen to the development of the high additional nutritional value functional food upsurge. The development of functional foods is generally achieved by adding food-grade bioactive ingredients (Figure 1), such as polyphenols [1], phytosterols [2], minerals [3], bioactive peptides [4], medications [5], and prebiotics [6]. However, due to their physical and chemical properties, many bioactive substances are often limited by poor stability, low water solubility, and low bioavailability, and thus cannot be introduced into the food system in a simple free state [7,8]. Therefore, adding micronutrients and bioactive substances to food formulations has been a major challenge and research focus in the food industry.

A common solution is to develop a delivery system for encapsulating, protecting, and controlling the release behavior of food-grade bioactive substances. A delivery system designed and prepared by micro or nanotechnology can enhance the stability, solubility, releasability, and bioavailability of bioactive substances by encapsulating them and providing strategies and theoretical bases for the application of bioactive substances in food [9,10]. In the pharmaceutical and biomedical fields, many polymers have been used to construct intelligent, regulatory, and selective drug delivery systems that have achieved the protection and transport of drug molecules to their targeted areas [11,12]. However, these materials are rarely used in foods because of their cytotoxicity or lack of biocompatibility and biodegradability. Food-grade delivery systems must be selected from a range of materials that have been granted safety certification. Presently, food-grade delivery materials that can encapsulate bioactive ingredients mainly include proteins, polysaccharides, and lipids, among others [13,14,15].

Among these food-grade materials, protein is a multifunctional bio-based polymer with rich nutritional value and various functional properties, including emulsification, amphiphilicity, gelation, and foaming. Its unique chemical structure and function enable it to be designed into nanoparticles, nanogels, nanoemulsions, nanofilms, and nanofibers, which allow the delivery of hydrophilic and hydrophobic bioactive components [16,17,18,19,20]. Plant protein is becoming increasingly popular as the material of choice for food-grade delivery systems, partly because of consumers’ growing interest in more economical and environmentally friendly diets, as well as the rise of vegetarianism and environmentalism in Western culture [21]. As a green and renewable resource, plant protein has gradually become a substitute for traditional animal protein in the food field. Scientists and food manufacturers are trying to understand how these plant proteins can partially or completely replace traditional animal protein to provide the best nutrition, flavor, and function. Recently, the function of plant proteins has been the focus of various studies [22]. The rise of plant milk, artificial meat, and plant yogurt, among others, further confirms this point. Additionally, recent studies have found that plant proteins may be more suitable than traditional animal proteins to develop into delivery vectors for bioactive substances [23,24]. For example, zein and gliadin can produce carriers that continuously release active substances, avoiding the use of toxic chemical crosslinkers and opening up new directions for the application of active substances in food systems [25,26]. The development of plant-based protein-delivery materials could provide consumers with new types of functional foods, such as energy drinks or plant-based meat [27,28]. Therefore, recent studies have focused on using plant proteins to construct food-grade natural carriers that will deliver various bioactive compounds in food.

In this study, we summarized the characteristics, advantages, and potential applications of different plant proteins, such as zein, soy protein, gluten, rice protein, etc. Moreover, the research progress on the different plant protein-based carriers in the encapsulation, protection, gastrointestinal tract delivery, and controlled release of bioactive substances was also reviewed. Green food-grade plant protein-based delivery systems, including nanoparticles, emulsions, films, gels, and fibers, will be expected to have great application potential in the functional food and biomedical fields.

## 2. Plant-Based Protein Properties

Compared to other natural macromolecules and synthetic polymers, proteins are amphiphilic, biocompatible, and biodegradable, and have many advantages in developing nanoscale bioactive substance-delivery systems [29,30]. Proteins are biological macromolecules composed of amino acids and have a very complex molecular structure. Although the side chain groups of amino acids afford proteins unique hydrophobicity, polarity, and charge, the complexity of proteins’ molecular structures allows nano-enabled carrier structures with different loading and releasing capacities in different processing methods to be obtained [31]. Furthermore, the unique structure of proteins allows them to be used in coating other biomaterials for targeted delivery [32]. Therefore, protein nano-enabled carriers hold great promise for loading and delivering plant polyphenols, essential oils, vitamins, and other biologically active substances. 

### 2.1. Zein

Zein is recognized as an environmentally friendly material and can be safely used in food [33]. It is produced as a co-product when corn grains are processed into food, agricultural products, and feed [34]. The average molecular weight of zein is about 40 kDa. Zein can be divided into α-zein, β-zein, γ-zein, and δ-zein. Structurally, zein contains many non-polar amino acids. The presence of many uncharged amino acid residues makes zein insoluble in water, but soluble in certain concentrations of ethanol, anionic surfactant, and alkaline solution (pH > 11), or a high-concentration urea solution. Zein has a wide source, low price, and is rich in many kinds of amino acids. Due to its good biocompatibility, biodegradability, self-assembly characteristics, film formation, and amphiphilicity, it has been extensively studied and applied in the food and pharmaceutical fields [35]. 

Recently, the advantages of zein as a natural macromolecular material applied as a carrier of bioactive substances have also attracted extensive attention. Macromolecular carriers can effectively improve the stability and bioavailability of active substances in vivo and have the advantage of enhancing the selectivity and slow release of bioactive substances [36]. Zein has a wide range of isoelectric points that are suitable for transporting different bioactive substances into the body. According to recent studies, the study of zein as a delivery carrier of bioactive substances primarily improves the stability and bioavailability of water-insoluble active substances. Thus, researchers designed nanoparticles and gels using zein as the carrier material nanofilm fiber. Furthermore, to reduce the toxic and side effects of bioactive substances on normal tissues and organs, and improve selectivity, researchers designed nano-delivery systems with targeted effects [37].

### 2.2. Soy Proteins

Beans are not only an excellent source of food protein, but are also among the most extensively used and consumed sources of protein. Legumes are rich in source, high in yield, low in cost, and low in sensitization, and have gradually become a substitute for animal protein in the food industry. Beans have rich nutritional value and probiotic effects, and can prevent coronary heart disease, diabetes, hypertension, and other diseases [38,39]. The unique particle size distribution, electric charge, emulsification, foaming, and amphiphilicity of legumes allow many applications in functional foods. Nanocapsules prepared by nanotechnology are widely used in the packaging, protection, and transportation of food-grade bioactive substances, which will be conducive to the functionalization of soy protein and to expanding and deepening the application of soy protein in the food field [40].

Soy protein, a co-product of soybean oil processing, is a spherical protein. Its fortified form is known as a soy protein isolate and has long-term storage stability. Soy protein contains polar, non-polar, and charged amino acid residues, including tyrosine, lysine, phenylalanine, leucine, glutamate, and aspartic acid, that can interact with various biologically active molecules and drugs, the most common interactions being hydrogen bonds, hydrophobic interactions, and van der Waals forces. In addition, soy protein has good biocompatibility, has been extensively used to prepare nanoparticles, hydrogels, and emulsions, and has broad application prospects in the functional food and biomedical fields [40]. In related studies, soybean protein-based biomaterials could induce the formation of new tissues, reduce the inflammatory response of host cells, and promote wound healing [41]. Soybean protein-based fiber has shown good antibacterial activity and biocompatibility, and can be used as a dressing for wound repair [42]. Furthermore, soybean protein nanocapsulators can be grafted onto targeted molecules to improve the bioavailability of the targeted molecules [43]. Finally, soybean protein can also be prepared into hydrogels for a controlled release system of bioactive substances to improve the bioavailability of the bioactive substances [16].

### 2.3. Glutenin and Gliadin

Wheat contains about 13% protein, which is mainly composed of albumin, globulin, gliadin, and glutenin, and wheat gluten protein mainly contains gliadin and glutenin. Glutenin has a molecular weight of about 106 kDa and is a protein insoluble in alcohol—its isoelectric point is 4.5~4.6. Due to the presence of a certain amount of disulfide cross-linked cysteine in the structure, it has good water stability and can be used for controlled release applications and tissue engineering in the form of nanoparticles, films, hydrogels, and fibers [17,44]. Gliadin has a molecular weight of 25~100 kDa and is a class of proteins isolated from alcohol. Gliadin is rich in proline and is almost insoluble in aqueous solutions—its isoelectric point is 6.41~7.1. Glutamine and hydrophobic amino acids in the gliadin structure enable gliadin to form hydrogen bonds with gastric mucosa and have hydrophobic interactions with the cell membrane, thus highly binding with the body’s mucosa. Because of their interaction with the gastric mucus, these nanoparticles increase the bioactivity of the active substance and the release time of the bioactive substance, thereby greatly improving the bioavailability of the bioactive substance. In addition, wheat gliadin films can also be used to control the release of bioactive substances, thus expanding their applications in the fields of food nutrition and tissue engineering [45].

### 2.4. Rice Proteins and Other Plant Proteins

Rice is one of the world’s major food crops and is now grown in more than 100 countries worldwide, with an annual production of over 40 billion tons. Rice protein has excellent qualities and its hypoallergenic nature makes it of higher biological value [46]. Additionally, rice protein is a good source of low fat, low cholesterol, and high protein. The nanocarriers prepared by using rice protein are not only economically low cost and green, but can also be broken down into active peptides by the action of digestive enzymes, which can play a variety of physiological roles in the body [47]. In view of this, the application of rice protein in nano drug-delivery systems has attracted extensive research by scholars. For example, Xu et al. successfully encapsulated and slow-release-delivered lutein using carboxymethyl cellulose compounded with rice protein, providing a new idea for the application of lutein in food [48]. In addition, some other plant proteins, such as nut proteins and peanut proteins, have been studied in the process of plant protein-based nano-delivery. They often have some functional properties that distinguish them from common plant proteins and can be used in some special food processing to meet specific needs.

## 3. Application of Different Types of Plant Protein-Based Nano-Enabled Carriers in the Encapsulation, Protection, and Delivery of Bioactive Components

**Figure 2:**
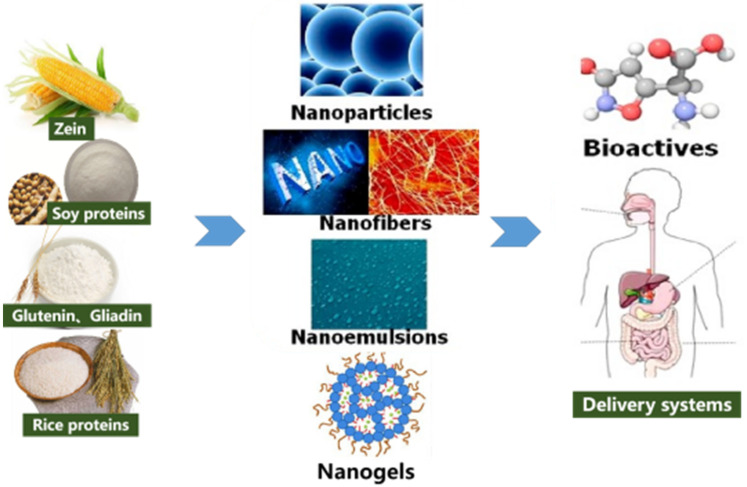
Nanostructured plant proteins: fabrication and applications as delivery systems for bioactives [40] (Reprinted with permission from Chuan-He Tang (2019). Copyright 2019 Elsevier).

Plant protein has become a potential candidate for bioactive material delivery because of its high load and excellent protective effect. Compared with traditional animal proteins, plant proteins have better hydrophobicity and water stability, are more stable in the water environment without diplomatic links, have a higher load capacity for hydrophobic bioactive substances, and have a longer-lasting payload release [49]. Furthermore, the use of plant proteins can reduce the risk of transmitting zoonotic diseases [50]. Therefore, the development of nano-enabled carriers, such as nanoparticles, gels, emulsions, and fibers, from highly cross-linked plant proteins may be a promising approach to improve the stability and bioavailability of various bioactive substances (Figure 2).

### 3.1. Plant Protein-Based Nanoparticles

Plant protein nanoparticles have been widely used for food-grade bioactive material delivery due to their biodegradability, biocompatibility, and easy surface modification. The application of nanotechnology in food, especially the development of bioactive food ingredients in functional foods with improved water solubility, physicochemical stability, absorptivity, and oral utilization, has led to the design of nanoparticles with specific sizes, structures, and functions. Given their small size, nanoparticles can penetrate the submucosal layer of tissues and be directly ingested by cells, resulting in better absorption and bioavailability [51]. The unique functional properties of plant protein-based nanoparticles afford them a higher loading capacity and slow-release effect in various nano-delivery systems. Some studies of plant protein-based nanoparticles for the encapsulation, protection, and delivery of bioactive substances are listed in Table 1.

Kim et al. [52] prepared zein nanoparticles using the liquid–liquid dispersion method and studied their encapsulation and efficiency on menthol. The experimental results showed that, when the concentration of menthol in 90% ethanol solution was greater than 5.4%, all of the menthol in the final solution was encapsulated, and the encapsulation efficiency was more than 90%. The optimal encapsulation conditions of menthol were obtained by changing the experimental parameters, and the optimum conditions were also applied to the encapsulation of other bioactive substances. Inchaurraga et al. [19] designed and prepared a zein nanoparticle-coated poly(anhydride)–thiamine coupling with a particle size of 250 nm and a negative surface charge for insulin encapsulation and delivery. The results showed that insulin loaded with zein nanoparticles had a better-sustained release effect than free insulin, and its inhibitory effect on the lipid metabolism of *C. elegans* under high glucose was about twice that of free insulin. Yuan et al. [55] designed curcumin-loaded Zein–CS–Spl ternary composite nanoparticles using zein, chondroitin sulfate (CS), and sophorolipid (Spl) as raw materials. The ternary composite system had a nanosphere structure and high zeta potential, which could encapsulate curcumin and other bioactive substances to achieve protection. When the ratio of curcumin to the ternary system was 1:1, it showed good anti-zein solubility, physical and chemical stability, and water solubility. Moreover, the ternary system had good biocompatibility, a bioavailability 68.06% higher than that of free substances, and anti-cancer activity. This study provided a new idea for designing a bioactive substance-packaging system and helped to further expand its application in functional foods. 

Bean protein is another raw plant protein material for preparing bioactive substance-delivery carriers. It also has good biocompatibility and bioaccessibility, rich economic sources, and unique functional properties, making it a hot spot for scholars’ attention and study. Zhang et al. [62] successfully prepared curcumin-supported soybean protein nano-composite particles with an average particle size of 244.7~344.7 nm using a self-assembled nano-complex reaction between curcumin and soybean protein. The fluorescence spectra results showed that the fluorescence intensity of the curcumin-loaded soy protein nanoparticles was decreased, the maximum loading of curcumin (9.97 μg/mg) was achieved when the degree of protein hydrolysis was 5%, and the storage stability was best. The results of this study provided a new reference and idea for the preparation, optimization, and selection of plant protein-delivery systems in functional foods. Wang et al. [63] used soybean protein isolate and sodium cellulose crystals as preparation materials and designed and prepared novel composite nanocapsules supported by curcumin based on the electrostatic interactions, hydrogen bonds, and hydrophobic interactions among curcumin, soybean protein isolate, and sodium carboxymethyl cellulose. The average particle size of the composite nanoparticles was 197.7 ± 0.2 nm, and they remained relatively stable under different pH levels (3~9), temperatures (30~90 ℃), and salt concentrations (0~40 mmol/L). The results of the in vitro gastrointestinal tract simulation showed that the encapsulation rate of curcumin in the composite nanoparticles was 88.3%, the release of curcumin in the stomach was minimal, and the bioactive substance was well protected. Thus, the composite nanosystem is a promising delivery system for hydrophobic bioactive substances. 

Other grain proteins, such as gliadin and rice protein, have been gradually used to prepare bioactive substance-delivery systems. For example, gliadin nanoparticles have an encapsulation rate of over 91% for curcumin, and curcumin–zein nanosystems have better thermal stability than curcumin alone [73]. The encapsulation rate of resveratrol in pea protein nanoparticles was 74.08%. The nanosystem had good physical and chemical stability, which could protect resveratrol from degradation, enhance its antioxidant activity, and be used as a functional delivery system of hydrophobic bioactive substances in the food field [65]. The β-carotene encapsulated by barley protein nanoparticles was spherical, with an average particle size of 351 nm, and showed no cytotoxicity. The uptake and transport of β-carotene were significantly increased (15%) after encapsulation, and the nanoparticles had a strong retention ability in rat jejunum, which increased the sustained release time of β-carotene and enhanced its bioavailability [75]. The solubility of lutein was significantly improved after being coated with rice protein nanoparticles, and the strong affinity enhanced the sustained release effect of lutein. The green and safe process provided a valuable reference for applying nutritionally fortified food, supplements, and drug delivery [78].

### 3.2. Plant Protein-Based Emulsions and Gels

Plant protein is an economical, safe, and nutritious emulsifier. Recently, it has been found that the complexes constructed by proteins and bioactive substances, such as curcumin and resveratrol, can aggregate at the oil/water interface and simultaneously display the surface activity of proteins and antioxidant activity of bioactive substances [79]. The plant protein-based emulsion system has a good encapsulation and delivery effect on fat-soluble bioactive substances. Furthermore, it can enhance the stability of bioactive substances, such as curcumin, resveratrol, and lutein, in the food system to improve their bioavailability. Gels are three-dimensional networks of physical or chemical cross-linked polymers that capture bioactive substances for protection and delivery. Plant protein gel is biocompatible, biodegradable, green, and non-toxic, and can facilitate the addition of bioactive substances to food. In addition, the plant protein-derived gel network is pH responsive and contains many acidic or alkaline groups, which can adapt to the human gastrointestinal tract environment to achieve the in vivo delivery of bioactive substances. Table 2 lists some studies on the loading and delivery of bioactive substances by common plant protein-based gels and emulsions. 

Qiu et al. [94] evaluated the antioxidant effect and bioavailability by combining gliadin and resveratrol to form a spherical structure complex and then adding the complex to the emulsion system. The results showed that the stability and antioxidant effect of resveratrol in the complex were well protected. The simulated gastrointestinal experiment proved that gliadin could effectively improve the stability and bioavailability of resveratrol and was a suitable delivery carrier for bioactive substances in food solutions and emulsions. Chen et al. [95] prepared the gliadin stabilized emulsion gel using a two-step method and explored the effects of co-encapsulation and the emulsion gel on (-)-Epigallocatechin-3-gallate (EGCG) and quercetin. The results showed that the hydrophilic bioactive substance EGCG and hydrophobic bioactive substance quercetin were encapsulated in the inner water phase (encapsulation rate 65.5%) and oil phase (97.2%), respectively. In vitro-simulated gastrointestinal tract experiments showed that the emulsion gel improved the stability of EGCG, enhanced the solubility of quercetin in gastrointestinal conditions, and increased the bioavailability of quercetin and EGCG by four and two times, respectively. Cheng et al. [44] selected gliadin nanoparticles and Pickering latex gel as a food-grade β-carotene delivery system to explore the changes in the bioavailability of β-carotene. The results showed that the stability of β-carotene in Pickering gel could improve the protective effect and bioavailability of β-carotene, thus providing a valuable carrier choice for the application of β-carotene in functional foods.

Nezhad-Mokhtari et al. [80] used zein as raw material and successfully developed a network of curcumin-loaded zein nanoparticles/aldehyde-modified guar gum/fibroin hydrogel, which improved the bioavailability of curcumin in trauma treatment. The results showed that the hydrogel network structure had a dense pore size and network structure, good thermal stability and biodegradability, and inhibitory activity against *Bacillus* and *E. coli*. Liu and Li et al. [84] prepared a novel ethanol-induced composite hydrogel using zein and propylene glycol alginate (PGA) as raw materials. The composite hydrogels showed stronger hardness and elasticity than PGA hydrogels in maintaining the release of curcumin under simulated gastrointestinal conditions. Hu et al. [85] reported a simple method for preparing hydrophilic and hydrophobic core–shell structures using pure natural food-grade proteins and polysaccharides based on a new principle of gel network-limiting anti-solvent precipitation. When the microgel beads prepared by hydrophilic polysaccharides were immersed in the hydrophobic protein solution, the protein layer on the surface of the microbeads was precipitated slowly and controllably by the anti-solvent. As a result, a hydrophilic and hydrophobic core–shell structure was formed. This method is suitable for various gel systems, controllable for size and shell thickness, and has good moisture resistance and slow release. Therefore, it has great application prospects in protecting the release and controlled release of unstable or hygroscopically active bioactive substances. The composite hydrogels prepared by Zhang et al. [91] using soybean protein isolate and carrageenan as raw materials were tough, uniform, and dense, and had slow-release properties. The rheology and microstructure analysis of the Monascus yellow pigment load composite hydrogel confirmed the dense, perseverant network structure. The study of composite gels for water-soluble bioactive substance-coating release provides a new train of thought and is conducive to certain biologically active substances needed to continue improving the development and application of functional food. 

### 3.3. Plant Protein-Based Films and Fibers

Fiber has many applications in the food and biomedical fields, such as controlling the delivery and release of bioactive ingredients. Nanoscale fiber materials can be prepared by electrospinning and solution spinning. Plant proteins, such as zein and soybean protein, have been widely used to prepare fiber and in the delivery and controlled release of bioactive substances (Table 3). For example, Fereydouni et al. [96] prepared zein fibers loaded with curcumin using electrospinning technology. Through the study of the physical and chemical properties of zein fibers, it was found that the higher the proportion of curcumin loading, the higher the release rate in vitro.

In addition, zein fiber can maintain the biological activity of curcumin, provide conditions for cell proliferation, and improve the bioavailability of curcumin. Zein fiber prepared by the same method can also be used to load and protect proanthocyanidins [97], (-)-Epigallocatechin Gallate (EGCG) [98], and carotenoids [100,101]. The results showed that zein had a certain degree of encapsulation and protective effect on different kinds of bioactive substances, and could maintain and stabilize the functional characteristics of the active substances and improve their bioavailability. 

Gliadin could also be formed into nanofibers to encapsulate and deliver bioactive substances. For example, the gliadin nanofibers prepared by Akman et al. [17] via electrospinning technology had an average diameter of about 258~375 nm and a uniform and smooth surface. X-ray diffraction patterns showed that the encapsulation efficiency of gliadin nanofibers increased with the increase in the curcumin content, indicating that the nanofibers could encapsulate most of the curcumin. In vitro simulation experiments showed that gliadin nanofibers loaded with curcumin could maintain curcumin’s free radical-scavenging ability and had a sustained release effect for curcumin. Additionally, the antioxidant and antibacterial activities of curcumin-loaded nanofibers were significantly improved compared with curcumin alone. Therefore, gliadin nanofibers can be developed into effective carriers of bioactive substances, such as curcumin, and have potential applications in functional food and other fields.

Plant proteins, such as soybean protein and zein, can also be formed into films to serve as carriers for the transport and controlled release of nutrients and bioactive substances (Table 3). Reddy et al. [104] explored the potential of soy protein isolate as a release system for natural antiproliferators through the preparation of soy protein isolate membranes. The successful addition of curcumin to the membrane system was confirmed by Fourier transform infrared spectroscopy (FTIR) and a scanning electron microscope (SEM). When the cross-linking agent content was 10%, the loading efficiency of curcumin was highest (68%). Through different kinetic release models, it was found that the membrane system could enhance the sustained release of curcumin, which confirmed the potential of the soybean protein isolate membrane in the controlled release of bioactive substances. Bisharat et al. [105] prepared a novel colonic delivery membrane using zein and acetylated hyperamylose to load, protect, and deliver to the colon by adding corresponding bioactive substances to the membrane structure. A study of paracetamol encapsulated in zein film showed that the six-hour drug release rate was only 14% in the in vitro simulation experiment, indicating that the delivery system could effectively enhance the sustained release effect of paracetamol. Therefore, the zein–starch mixed membrane is a potential new bioactive substance delivery system that is conducive to expanding the application of bioactive substances in the nutritive food, functional food, and biomedical fields.

## 4. Conclusions

The most common bioactive substances, such as curcumin, catechin, quercetin, resveratrol, rutin, essential oils, lutein, and carotene, have poor biological water solubility, poor stability, and low bioavailability. These characteristics impose some limitations on their applications in areas such as food and biomedicine. Nevertheless, plant protein carriers are rich in raw materials and can be obtained economically and environmentally, which can protect the stability of sensitive bioactive substances and maintain their many beneficial properties. In addition, the good biocompatibility, biodegradability, environmental safety, and other characteristics of plant protein materials enable them to be used as a food-grade formula in functional food, beverages, and other fields. This study reviewed the application of plant protein delivery systems in the encapsulation, protection, and controlled release of bioactive components. Different forms of nano-enabled carriers have expanded the use of bioactive substances in food additives and functional foods. Moreover, compared to traditional animal proteins, plant proteins (mainly soy protein, zein, gluten, and rice bran protein) are more resource-rich and environmentally friendly, and can be designed and manufactured into nanoparticles, emulsions, films, gels, and fibers using nanotechnology. This is expected to provide a valuable reference for the development of novel green food-grade delivery systems, and, in the future, it is expected that plant protein-based nano-systems with multiple functional properties, such as functional ingredient delivery and interfacial stabilization of food dispersions (e.g., emulsions and gels), will be designed, providing new ideas and insights for the application of bioactive substances and plant proteins in the field of functional foods.

## Figures and Tables

**Table 1:** Plant protein-based nanoparticles for bioactive ingredient delivery.

Type of Nanoparticles	Preparation Method	Encapsulated Bioactives	Encapsulation Efficiency	Major Outcomes	Refs
Zein nanoparticles	Liquid–liquid dispersion method	Menthol	>90%	A feasible encapsulation carrier was designed for bioactive substances soluble in 90% ethanol.	[52]
Zein nanoparticles	Liquid antisolvent precipitation	Hibiscus sabdariffa extract	89%	At the same time, high encapsulation efficiency and good particle size control in the nanometer range were obtained.	[53]
Zein nanoparticles	Desolvation procedure	Insulin	8% (payload)	The pharmacological activity and relative availability of insulin were significantly improved after insulin was loaded with zein nanoparticles.	[19]
Zein nanoparticles	Electrospraying	Gallic acid	-	The preparation of zein gallic acid nanoparticles by the electrospray method was a feasible technology, which had a potential protective effect on gallic acid.	[54]
Zein nanoparticles	Nanoprecipitation method	Rutin	~88%	Zein nanosystem improved the stability and controlled release of rutin.	[25]
Zein–chondroitin sulfate–sophorolipid composite nanoparticles	Self-assembly technology	Curcumin	63.4% to 98.21%	The ternary nanocrystalline delivery system had good biocompatibility and provided a new idea for the delivery of bioactive substances.	[55]
Zein–propylene glycol alginate–rhamnolipid complex nanoparticles	Emulsification–evaporation method	Resveratrol; Coenzyme Q10	49.54 ± 4.37% to 91.80 ± 4.62%; 84.06 ± 1.49% to 95.51 ± 0.61%	The co-transfer of resveratrol and coenzyme Q10 was achieved, and the chemical stability and synergistic sustained release of resveratrol and coenzyme Q10 were improved.	[56]
Brij-stabilized zein nanoparticles	Nanoprecipitation technique	Rhodamine B; Bromophenol blue	~40%; ~80%	Brij-stabilized zein nanosystem prolonged the release time of the active compound and was a promising and innovative nanomaterial.	[57]
Alginate/chitosan-coated zein nanoparticles	Electrostatic deposition technique	Resveratrol	>70%	The alginate–chitosan layer significantly promoted the release and bioavailability of resveratrol in zein nanoparticles.	[58]
Zein/carboxymethyl dextrin nanoparticles	Antisolvent precipitation	Curcumin	85.5%	The nanoparticles significantly enhanced the photochemical stability, thermal stability, antioxidant activity, and gastrointestinal slow-release effect of curcumin.	[59]
Zein/soluble soybean polysaccharide composite nanoparticles	Antisolvent precipitation method	Lutein	>80%	The complex system was a promising lutein delivery system that could be added as an ingredient to beverages or functional foods.	[60]
Soy protein nanoparticles	Alkali soluble acid precipitation	Anthocyanin	90.02 ± 0.04% to 94.18 ± 0.04%	It provided a valuable reference for the preparation of a new type of Pickering emulsion and improved the stability of bioactive substances.	[61]
Soy protein nanoparticles	Self-assembled nanocomplexation	Curcumin	-	The 5% hydrolyzed soybean protein had the highest loading capacity for curcumin, relatively small particle size, and the best storage stability.	[62]
Soy protein isolate/cellulose nanocrystal composite nanoparticles	Self-assembly technology	Curcumin	88.3%	Composite nanoparticles had high encapsulation efficiency and slow release effect, and were a promising delivery carrier for hydrophobic bioactive substances.	[63]
Soybean protein isolate and fucoidan nanoparticles	Electrostatic interaction	Curcumin	>95%	The composite nanoparticles had a spherical core–shell structure, the embedding rate of curcumin could reach 95%, and the system had long-term dispersion stability.	[64]
Pea protein nanoparticles	Calcium-induced cross-linking	Resveratrol	74.08%	The nanoparticles could be efficient, powerful nanocarriers for the delivery of hydrophobic polyphenols, with great potential in functional beverages.	[65]
Grass pea protein isolate/Alyssum homolocarpum seed gum complex nanoparticles	Antisolvent precipitation	Curcumin	88.22%	The particles could delay the release of Cur under in vitro gastrointestinal conditions.	[66]
Core–shell pea protein–carboxymethylated corn fiber gum composite nanoparticles	Liquid–liquid dispersion method	Curcumin	99.2 ± 0.8% (pH = 3.5)	The core–shell structure afforded curcumin higher antioxidant activity, which provided a new strategy for the delivery of unstable hydrophobic active substances.	[67]
Peanut protein nanoparticles	Calcium-induced	Resveratrol	82.7%	This resveratrol-loaded PPN could serve as a promising delivery system for long-term anti-cancer.	[68]
Peanut protein nanoparticles	Ultrasound-assisted thermo–alkali modification	Curcumin	83.27 ± 1.06%	Compared with pure curcumin, the antioxidant activity was increased with the presence of peanut protein nanoparticles.	[69]
Peanut protein nanoparticles	Alkali extraction and acid precipitation methods	5-demethylnobiletin	-	It provided a new delivery strategy for 5-demethylnobiletin in functional food and beverages.	[20]
Walnut protein nanoparticles	Electrospray technique	Curcumin	61.45 ± 1.61%	The nanosystem could be used as a unique food-grade carrier to improve the water solubility and sustained release of curcumin.	[70]
Gliadin nanoparticles	Antisolvent precipitation	Resveratrol	68.2%	The stability, solubility, and antioxidant capacity of resveratrol were improved by the combination of gliadin nanoparticles and gum Arabic.	[26]
Gliadin–chitosan composite nanoparticles	Antisolvent precipitation	Curcumin	86.1%	The chitosan-modified gliadin nanoparticles showed higher encapsulation efficiency, better stability, and stronger antioxidant capacity for curcumin.	[71]
Gliadin–lecithin composite nanoparticles	Antisolvent precipitation	Curcumin	90.7 ± 0.3%	Gliadin–lecithin composite nanoparticles possessed higher encapsulation efficiency, better stability, and higher antioxidant activity.	[72]
Gliadin nanoparticles	Antisolvent precipitation	Curcumin	91%	Deaminated gliadin nanoparticles had a good encapsulation and protection effect on curcumin and had a good application prospect in the field of nutrition transmission.	[73]
Gliadin–rhamnolipid composite nanoparticles	pH-driven method	Curcumin	98.70%	Composite nanoparticles prepared by pH-driven phytic acid had the potential to be a good nanoparticle delivery system for curcumin in functional foods.	[74]
Barley protein nanoparticles	High-pressure homogenizing method	β-carotene	-	Barley protein nanoparticles could improve the adsorption performance and may be used as a carrier of hydrophobic compounds.	[75]
Rice bran albumin nanoparticles	Antisolvent precipitation approach	Curcumin	95.94%	Nanoparticulate curcumin formulation showed improved in vitro antioxidant activity, anti-inflammatory activity, and in vitro antiproliferative activity on tumor cells of curcumin in aqueous solution as compared with free curcumin.	[76]
Rice bran albumin–chitosan nanoparticles	Self-assembly technology	Curcumin	93.56%	Composite nanoparticles had good biodegradability and had great potential as green and renewable materials in the transport of hydrophobic active substances.	[77]
Rice protein	Antisolvent method	Lutein	89.8% to 94.1%	It provided a reference strategy for the stabilization of lutein and nutrient delivery.	[78]
Carboxymethylcellulose-modified rice protein nanoparticles	Antisolvent method	Lutein	-	This nano-system enhanced the absorption of lutein, which is helpful for the further development and application of new nano-delivery systems of lutein.	[48]

**Table 2:** Plant protein-based gels and emulsions for bioactive ingredient delivery.

Type of Gels	Encapsulated Bioactives	Encapsulation Efficiency	Major Outcomes	Refs
Zein hydrogels	Curcumin	-	The hydrogel network structure supported by curcumin had good biocompatibility, degradability, low cytotoxicity, and antibacterial properties.	[80]
Zein–co-acrylic acid hybrid hydrogels	Rutin	81.47%	The hydrogel structure had good stability, high encapsulation rate, and drug loading capacity for rutin, and was a good carrier of bioactive substances.	[81]
Zein thermosensitive gel	Lutein	95.9 ± 3.2%	The thermal gel had a good loading effect on lutein and improved the bioavailability of lutein.	[82]
Zein-based oil-in-glycerol emulgels	β-carotene	-	Zein-based oil-in-glycerol emulgels enhanced the stability and bioavailability of beta carotene.	[83]
Zein ethanol-induced composite hydrogel	Curcumin	-	The composite hydrogels had a good ability to maintain curcumin release under simulated gastrointestinal conditions.	[84]
Zein hydrophilic–hydrophobic core shell hydrogel	Bioactives	-	A general method for hydrophilic and hydrophobic core–shell hydrogels was designed to provide a new carrier for the delivery and controlled release of bioactive substances.	[85]
Zein and sodium alginate double cross-linked emulsion gels	Curcumin; Resveratrol	- -	The double cross-linked emulsion gels afforded higher light stability and bioaccessibility than the single-cross-linked ones.	[86]
Zein nano-emulsion	Lutein	-	Zein stable nanoemulsions had good protective and release effects on lutein, providing valuable insights for improving the bioavailability of fat-soluble bioactive substances.	[87]
Soy protein isolate and sugar beet pectin interpenetrating polymer network hydrogels	Probiotics	88.9%	The probiotics achieved better storage stability and higher vitality after loading the gel structure.	[88]
Soy glycinin gel-like emulsions	β-carotene	-	Gelatinous network slowed down the release of beta carotene and improved its bioavailability.	[89]
Soy protein cold-set emulsion filled gels	Curcumin	-	A new method was proposed to improve the stability of curcumin in the soybean protein gel system.	[90]
Soy protein isolate/κ-carrageenan composite hydrogels	Monascus yellow	-	The monaskoid yellow pigment in hydrogel system had higher stability and a better sustained release effect.	[91]
Soy protein isolate -Pleurotus eryngii polysaccharide conjugate-stabilized emulsion	β-carotene	-	Conjugated emulsion system was helpful to enhance the bioavailability of β -carotene and had broad application prospects in the transportation of fat-soluble nutrients.	[92]
Soy protein isolate emulsion	Lipophilic bioactive substance	86.97% to 91.68%	A new method for preparing functional Pickering emulsion as a transfer medium for functional lipophilic raw materials was provided.	[93]
Gliadin nanoparticles Pickering emulgels	β-carotene	-	Gliadin nanoparticle Pickering emulgels could improve the stability and bioavailability of β-carotene.	[44]
Gliadin emulsion system	Resveratrol	-	The emulsion system based on gliadin had a good encapsulation and delivery effect on resveratrol, which provided a valuable reference for the application of bioactive substances in the food system.	[94]
Gliadin emulsion gels	EGCG; Quercetin	65.5%; 97.2%	The emulsion gel increased the chemical stability and solubility of quercetin in simulated gastrointestinal tract conditions, and the effective bioaccessibility of quercetin was increased by four times.	[95]

**Table 3:** Plant protein-based fibers and films for bioactive ingredient delivery.

Type of Material	Preparation Method	Encapsulated Bioactives	Encapsulation Efficiency	Major Outcomes	Refs
Zein fibers	Electrospinning technique	Curcumin	94% ± 3.04%	Curcumin was successfully woven into zein fibers and maintained its functional properties.	[96]
Zein fibers	Electrospinning technique	Proanthocyanidins	close to 100%	Zein–proanthocyanidin fiber had good controlled release effect on proanthocyanidin.	[97]
Zein fibers	Continuous electrospinning	EGCG	-	EGCG was encapsulated in zein fiber, which provided a new strategy for biological delivery of EGCG.	[98]
Zein fibers	Electrospinning technique	Curcumin	close to 100%	The encapsulation rate of curcumin was close to 100%, and the good antioxidant activity and bacteriostasis were retained after encapsulation.	[99]
Zein fibers	Electrospinning technique	Carotenoids	>90%	Zein fiber had a good stabilizing effect on carotenoids and had a broad application prospect in the production of functional food.	[100]
Zein fibers	Electrospinning technique	Carotenoids	>77%	Good encapsulation of carotenoids and an ideal release effect of carotenoids in gastrointestinal tract were achieved.	[101]
Zein–chitosan composite electrospun fibers	Electrospinning	α-tocopherol	-	Composite electrostatic fibers had good adhesion and release properties in gastric mucosa, and had potential applicability in the transport of hydrophobic compounds to the gastrointestinal tract.	[102]
Zein ultrafine fibers	Electrospinning	Folic acid	>80%	The folic acid stability of the zein packaging was improved, which was beneficial to expand its application in functional foods.	[103]
Gliadin nanofiber	Electrospinning	Curcumin	80% to 85%	The inclusion of curcumin in nanofibers significantly enhanced their antioxidant and antibacterial activities, and gliadin nanofibers had potential applications in the food industry and other biological activity delivery systems.	[17]
Pea protein core–shell composite fiber	-	Curcumin	99.2 ± 0.8%	The core–shell composite nanomaterial showed excellent encapsulation performance and stability, and showed higher antioxidant activity and free radical-scavenging ability than free curcumin.	[67]
Soy protein isolate films	Casting method	Curcumin	42% to 68%	Soybean protein isolate membrane had higher curcumin loading capacity and improved the bioavailability of curcumin.	[104]
Zein–high-amylose starch films	-	Paracetamol	-	Zein membrane could achieve colon-targeted drug delivery, which provided a feasible strategy for improving the bioavailability of bioactive substances.	[105]
Zein–starch composite film	-	Clove essential oil	-	The compound membrane had a higher loading rate and better release effect on clove essential oil.	[18]

## Data Availability

Not applicable.
